# What Are the Human Resources Required to Control a Foot-and-Mouth Disease Outbreak in Austria?

**DOI:** 10.3389/fvets.2021.727209

**Published:** 2021-10-28

**Authors:** Tatiana Marschik, Ian Kopacka, Simon Stockreiter, Friedrich Schmoll, Jörg Hiesel, Andrea Höflechner-Pöltl, Annemarie Käsbohrer, Beate Conrady

**Affiliations:** ^1^Unit of Veterinary Public Health and Epidemiology, Institute of Food Safety, Food Technology and Veterinary Public Health, University of Veterinary Medicine, Vienna, Austria; ^2^Division for Animal Health, Austrian Agency for Health and Food Safety, Mödling, Austria; ^3^Division for Data, Statistics and Risk Assessment, Austrian Agency for Health and Food Safety, Graz, Austria; ^4^Department for Animal Health and Animal Disease Control, Federal Ministry of Labour, Social Affairs, Health and Consumer Protection, Vienna, Austria; ^5^Department of Veterinary Administration, Styrian Provincial Government, Graz, Austria; ^6^Department of Veterinary and Animal Sciences, Faculty of Health and Medical Sciences, University of Copenhagen, Copenhagen, Denmark; ^7^Complexity Science Hub, Vienna, Austria

**Keywords:** contingency planning, EuFMDiS simulation model, foot-and-mouth disease, preparedness activities, resource planning

## Abstract

Contingency planning allows veterinary authorities to prepare a rapid response in the event of a disease outbreak. A recently published foot-and-mouth disease (FMD) simulation study indicated concerns whether capacity was sufficient to control a potential FMD epidemic in Austria. The objectives of the study presented here were to estimate the human resources required to implement FMD control measures and to identify areas of the operational activities that could potentially delay successful control of the disease. The stochastic spatial simulation model EuFMDiS (The European Foot-and-Mouth Disease Spread Model) was used to simulate a potential FMD outbreak and its economic impact, including different control scenarios based on variations of culling, vaccination, and pre-emptive depopulation. In this context, the utilization of human resources was assessed based on the associated EuFMDiS output regarding the performance of operational activities. The assessments show that the number of personnel needed in an outbreak with a stamping-out policy would reach the peak at the end of the second week of control with a median of 540 (257–926) individuals, out of which 31% would be veterinarians. Approximately 58% of these human resources would be attributable to surveillance, followed by staff for cleaning and disinfection activities. Our analysis demonstrates that, of the operational activities, surveillance personnel were the largest factor influencing the magnitude of the outbreak. The aim of the assessment presented here is to assist veterinary authorities in the contingency planning of required human resources to respond effectively to an outbreak of animal diseases such as FMD.

## Introduction

Foot-and-mouth disease (FMD) is a highly contagious transboundary viral disease, which, under certain conditions, may have a large economic and social impact on the livestock industry and the economy as a whole (1–6). Potential consequences of an FMD epidemic include the loss of international market access, disruption of the domestic market for livestock animals and related animal products, serious production and income losses for livestock owners, and considerable costs of eradicating animal disease (7).

Disease spread simulation models provide valuable insights into the dynamics and course of the epidemic, especially in countries that have not recently experienced an FMD outbreak (1–4, 8) such as Austria. In the previous study by Marschik et al. (1), the European Simulation Disease Spread Model (EuFMDiS) (9) was used to evaluate the epidemiological and economic impact of a potential FMD outbreak in Austria. According to these estimates, the costs and losses of an FMD outbreak in Austria would range from € 269 million to € 581 million, depending on the region affected and the control measures implemented. The results indicated, that limited availability of human resources is a serious constraint to the rapid and effective implementation of control measures and may have a negative impact on the magnitude of the outbreak. Marschik et al. (1) assumed that increasing the availability of human resources may limit the size of the epidemic and reduce the economic losses to a degree comparable to the effect of additional control measures such as vaccination or pre-emptive culling compared with the stamping-out policy.

Only a limited number of FMD simulation models incorporate the human resources component in order to allocate resources for control of an outbreak. Such models, including EuFMDiS, are capable of evaluating whether human resources for operational activities such as surveillance, culling, disposal, and/or vaccination are sufficient to eradicate the epidemic without delay and/or whether additional resources are required (1, 6, 9). The FMD outbreaks in the United Kingdom (UK) (10), the Netherlands (11), and Japan (12) have identified several critical bottlenecks in resource adequacy [e.g., in number of required staff to perform the operational activities (13)], which were major obstacles in the disease control efforts. Whether sufficient resources are available in case of an animal disease outbreak is essential for the contingency planning of veterinary public authorities.

The aims of this study presented here were (i) to identify areas of operational activities where the currently available human resources in Austria may be insufficient, (ii) to estimate the overall human resources needed to respond time-efficiently to an FMD outbreak, and (iii) to assess how the availability of resources impact the overall losses in case of an FMD outbreak in Austria.

## Materials and Methods

In contrast to the study published by Marschik et al. (1) which simulated the FMD outbreak in Austria with limited human resources to perform the operational activities, the study presented here used an approach with unlimited human resources. Resource utilization reports from both the limited and unlimited human resource approaches were analyzed to estimate the resources required to effectively control FMD. In this context, the areas of operational activities which constrained the effectiveness of control measures were identified. Further, the information regarding daily, and total numbers of premises undergoing each operational activity were converted in daily staff requirements.

### EuFMDiS Application in Austria

In a previously published study by Marschik et al. the EuFMDiS Model (version 1.6.) was used to simulate a hypothetical outbreak of FMD in Austria (1). In brief, EuFMDiS considers spread of disease within and between herds. The latter includes five different pathways: (i) direct contact, (ii) indirect contact, (iii) local spread, (iv) airborne transmission, and (v) assembly centers. The model is configured to support the control measures established in the European FMD legislation (14), i.e., (i) detection of first infected farm, (ii) movement restrictions, (iii) reporting of suspected farms, (iv) surveillance visits, (v) tracing, (vi) operational activities in infected farms (i.e., culling, pre-emptive culling, disposal, cleaning, and disinfection), and (vii) vaccination [suppressive ring vaccination (i.e., “vaccination to kill,” carried out within the infected areas in order to reduce virus shedding; it is assumed that infection is present, and when time and resources permit, these animals will be slaughtered) or protective ring vaccination (i.e., “vaccination to live,” carried out outside known infected areas in order to protect susceptible animals from infection)] (15).

The susceptible Austrian livestock population [i.e., 5.32 million susceptible animals (51,014 cattle herds, 19,184 swine herds, 17,279 sheep and goat herds, and 19,190 backyard herds)] was categorized into nine farm types and eight herd types in the simulation model, based on the type of species, herd size, and production system [see detailed description in the study by Marschik et al. (1)].

The hypothetical outbreak of FMD was initiated in a livestock-dense region (96 livestock animals/km^2^) in Austria. This region, which is characterized by an intensive livestock production (i.e., 58% of FMD-susceptible Austrian livestock population) and a number of large cattle and swine herds [i.e., median (25^th^ and 75^th^ percentiles) of cattle herd size: 34 (20–58) and swine herd size: 15 (2–299)] as well as a high rate of animal movements.

In line with the previous study by Marschik et al. (1), the outbreak was initiated in a randomly chosen dairy cattle herd. In each iteration (1,000 per simulation) a different dairy herd was randomly selected in order to consider the variation of herd size and movement patterns in the chosen region. The same 1,000 seed herds were used in all implemented control strategies to ensure comparability of the effect of individual control measures. It was assumed that the outbreak was not detected for 21 days (referred to as silent phase). The implementation of control measures started after the silent phase (referred to as control phase). Each simulation was run until the disease was eradicated or up to 365 days if the outbreak was not controlled.

In this study, five different control scenarios were compared: (i) reference scenario (SO) (i.e., stamping out of all infected herds), (ii) pre-emptive depopulation of dangerous contact herds (SODC) (i.e., reference scenario and stamping out of dangerous contact herds based on tracing movements of livestock and its products), (iii) pre-emptive depopulation of susceptible herds (SORC1) (i.e., reference scenario and stamping out of all susceptible herds within a 1 km radius around infected herds), (iv) suppressive vaccination (SOSV1) (i.e., stamping out of infected herds plus suppressive ring vaccination within 1 km radius of infected herds), and (v) protective vaccination (SOPV1) (i.e., stamping out of infected herds plus protective ring vaccination within 1 km radius of infected herds). The epidemiological and economic magnitudes of the outbreaks were compared between all simulated scenarios, and the optimal control strategy was considered to be the one with the lowest total cost. The epidemiological indicators compared included the number of infected farms, the number of depopulated farms and animals, and the epidemic control duration. Wilcoxon rank sum test was used to test the statistical differences between the scenarios with limited and unlimited resources.

### Resource Approach in EuFMDiS

EuFMDiS models disease control as a resource-intensive process and a shortage in available personnel can hamper the simulated outbreak response (9). The resource capacity is defined in the EuFMDiS configuration data and is organized into pools of teams for five operational key activities: surveillance, culling, disposal, cleaning and disinfection, and vaccination. The teams are deployed during the control phase of the outbreak in accordance with the magnitude of the outbreak and the control measures chosen. The number of teams can be adjusted in the model to match the available personnel required to perform each operational activity. The number of available teams is assumed to increase over the control period, i.e., initially, the available team pool is small and increases linearly to a predefined maximum size. When an operational activity is scheduled and a resource is available, it is taken from the corresponding pool. However, if the resource capacity is insufficient at a point in time, the activity is queued until resources become available. This can result in prolonged control duration and/or larger outbreaks. Resources are returned to the pools once the activity is complete. The model also provides the option of not limiting the pools, i.e., teams are always immediately available upon request (9).

The surveillance component of EuFMDiS carries out visits to all herds in protection zones and to contact, suspect, and trace herds in surveillance and free zones (9). Surveillance visits are prioritized according to the risk of infection, which depends on the herd classification (e.g., suspect, trace, or contact herd) and the control zones (e.g., protection, surveillance, and free zone). EuFMDiS maintains resource-constrained prioritized queues of holdings awaiting a surveillance visit. If multiple herds have the same priority, then arbitration is based on how long a herd has been waiting for a visit. The visit duration (based on herd type), visit frequency (based on priority), and overall surveillance period are configurable in the model. Further information on how surveillance is modeled in EuFMDiS can be obtained from Bradhurst et al. (9). EuFMDiS provides information regarding resource utilization in terms of daily and total numbers of premises undergoing various operational activities, including backlogs during the control phase of the outbreak. These reports are available for surveillance, culling, disposal, and vaccination. Resource indicators, such as the number of conducted surveillance visits and the number of overall operational activities were compared between the conducted scenarios.

To identify the areas of FMD operational activities where currently available human resources in Austria may be insufficient, analyses of daily resource utilization reports from the simulations in the study by Marschik et al. (1) were conducted. Furthermore, the same simulations were performed with unlimited resources. The latter approach provides information on an optimal epidemiological situation and thus influences the economic results. The daily and total numbers of premises undergoing each operational activity were converted into staff requirements, where two types of parameters were taken into account: (i) time required for a herd to undergo each operational activity based on herd type and (ii) size of team required for each operational activity. The time requirements for the operational activities and the composition of the required team were estimated by experts from the Austrian Federal Ministry of Social Affairs, Health, Care and Consumer Protection, the Austrian Agency for Health and Food Safety, and the University of Veterinary Medicine Vienna (Supplementary Table 1) based on the knowledge and experience from other animal diseases and are in line with the scientific literature (6, 7, 16). During an outbreak, additional personnel is required for management, administration, logistics, tracing, training, communication with media, etc., in a national disease coordination center and local disease crisis centers. The activities associated with cleaning and disinfection of detected herds are the responsibility of veterinary authorities with the support of the Austrian Armed Forces. In the study presented here, all required human resources in an FMD outbreak were distinguished between veterinarians and “others.” “Others” are defined as staff which support veterinarians during the operational visits and/or are responsible for administrative and logistical tasks in the coordination centers (Supplementary Table 1).

To assess how the availability of resources impacts the overall economic losses of an FMD outbreak in Austria, an assessment of the associated costs and losses were performed. These include direct costs and indirect costs. The methodology of the cost assessment was described in the previous study by Marschik et al. (1). In brief, direct losses cover costs of control activities (including compensation payments) and were calculated by the EuFMDiS model (Supplementary Table 2). Indirect costs were estimated by our own economic approach and included the following costs: export losses (i.e., losses due to export bans on livestock animal and livestock products), production losses in zones (i.e., production losses for the farmers resulting from business interruption due to movement restrictions within protection and surveillance zones) and production losses in culled herds (i.e., losses resulting from the temporary vacancy of stables for owners, whose herds were culled) (Supplementary Table 3).

## Results

In the simulations with limited resources (Supplementary Figure 1), insufficient availability of surveillance personnel was found to limit the effectiveness of control measures in all scenarios. In the reference scenario (SO), the epidemic had a median size (25^th^-75^th^ percentiles) of 81 (27–183) infected herds and an epidemic control duration of 76 (46–128) days (Table 1). The number of farms that were subject to surveillance exceeded surveillance capacity immediately after detection (day 23) and this bottleneck lasted for 41 (21–74) days. In the median, there were 411 (138–1,067) pending surveillance visits per day during the control phase. Personnel for cleaning and disinfection was insufficient only in the scenario where pre-emptive depopulation was applied (SORC1). In this scenario, 284 (126–587) herds were depopulated and thus subject to cleaning and disinfection, which lead to prolonged period between the last day of culling, namely 57 (45–74) and the last day of control, namely 171 (93–326). The limited resources for culling, disposal, and vaccination (Supplementary Table 1) were sufficient in all considered scenarios and did not constrain the effectiveness of the respective implemented measure during the epidemic control phase.

**Table 1:** Comparison of epidemiological and economic results of simulations with limited and unlimited human resources.

**Scenario^a^**	**Resources**	**Infected farms**	**Depopulated farms**	**Depopulated animals**	**Last day of culling**	**Last day of control**	**Epidemic control duration^b^ (days)**	**Vaccinated farms**	**Direct costs (in Mio. €)**	**Indirect cost (in Mio. €)**	**Total cost (in Mio. €)**
SO	Limited	81 (27–183)	81 (27–183)	4,924 (1,687–11,454)	80 (52–118)	97 (67–149)	76 (46–128)	–	24 (10–47)	519 (246–869)	543 (255–915)
	Unlimited	61 (27–102)	61 (27–102)	3,616 (1,477–6,633)	50 (42–58)	65 (57–73)	44 (36–52)	–	25 (11–42)	413 (232–518)	437 (232–518)
SODC	Limited	73 (28–172)	76 (29–175)	4,683 (1,661–11,175)	77 (51–117)	94 (66–151)	73 (45–130)	–	23 (10–45)	513 (244–857)	536 (254–901)
	Unlimited	54 (23–97)	56 (24–100)	3,499 (1,357–6,582)	49 (41–58)	64 (56–73)	43 (35–52)	–	26 (1–45)	397 (232–511)	419 (242–550)
SORC1	Limited	40 (19–80)	284 (126–587)	15,422 (5,961–33,092)	57 (45–74)	171 (93–326)	150 (72–305)	–	26 (12–51)	433 (238–633)	460 (250–683)
	Unlimited	40 (19–72)	246 (112–456)	14,175 (5,690–27,898)	48 (40–57)	63 (55–72)	42 (34–51)	–	26 (12–47)	400 (231–518)	425 (243–565)
SOSV1	Limited	66 (26–140)	66 (26–140)	4,136 (1,500–9,296)	69 (51–94)	84 (66–121)	63 (45–100)	169 (62–373)	20 (9–38)	461 (244–688)	481 (254–725)
	Unlimited	53 (24–96)	53 (24–96)	3,488 (1,455–6,221)	53 (44–64)	68 (59–79)	47 (38–58)	139 (55–247)	21 (9–37)	403 (235–515)	425 (244–552)
SOPV1	Limited	68 (26–133)	68 (26–133)	4,135 (1,601–8,661)	69 (52–93)	84 (67–118)	63 (46–97)	171 (65–348)	20 (9–36)	561 (341–782)	581 (350–819)
	Unlimited	54 (24–95)	54 (24–95)	3,358 (1,398–6,353)	53 (44–63)	68 (59–78)	47 (38–57)	134 (56–244)	21 (9–37)	498 (331–603)	519 (340–640)

*All conducted outbreak scenarios were initiated in the same index herds (1,000 herds per simulation) and the model results are presented as median (25th and 75th percentiles)*.

a *The different control measures are: SO, stamping out of all infected herds (reference scenario); SODC, pre-emptive depopulation of dangerous contact herds; SORC1, pre-emptive depopulation of all susceptible herds within 1-km radius around infected herds; SOSV1, suppressive vaccination of all susceptible herds with 1-km radius around infected herds; SOPV1, protective vaccination of all susceptible herds with 1-km radius around infected herds*.

b *Epidemic control duration is calculated from the detection of the first infected herd (day 21) to the day of lifting of the last restricted zone (i.e., last day of control)*.

Comparison of the results of simulations with limited and unlimited resources showed the negative impact of constrained availability of resources on the epidemic magnitude and the overall costs (Table 1). Assuming no upper limit on resource capacity, the number of infected farms in the reference scenario would decrease by 25% and the total cost by 19%. In the pre-emptive depopulation control strategy, unlimited resources offered no benefits in terms of outbreak size but decreased the overall cost by 8%. Except for this strategy, all control strategies with unlimited resources were significantly different on the 0.05 level compared to the scenarios with limited resources in terms of infected farms, epidemic control duration, and total cost. Among all control strategies with unlimited response capacity, implementation of the depopulation of dangerous contact herds (SODC) generated the lowest total cost of € 419 (242–550) million, while the protective vaccination control strategy (SOPV1) resulted in the highest median total cost of € 581 (350–819) million.

**Figure 1:**
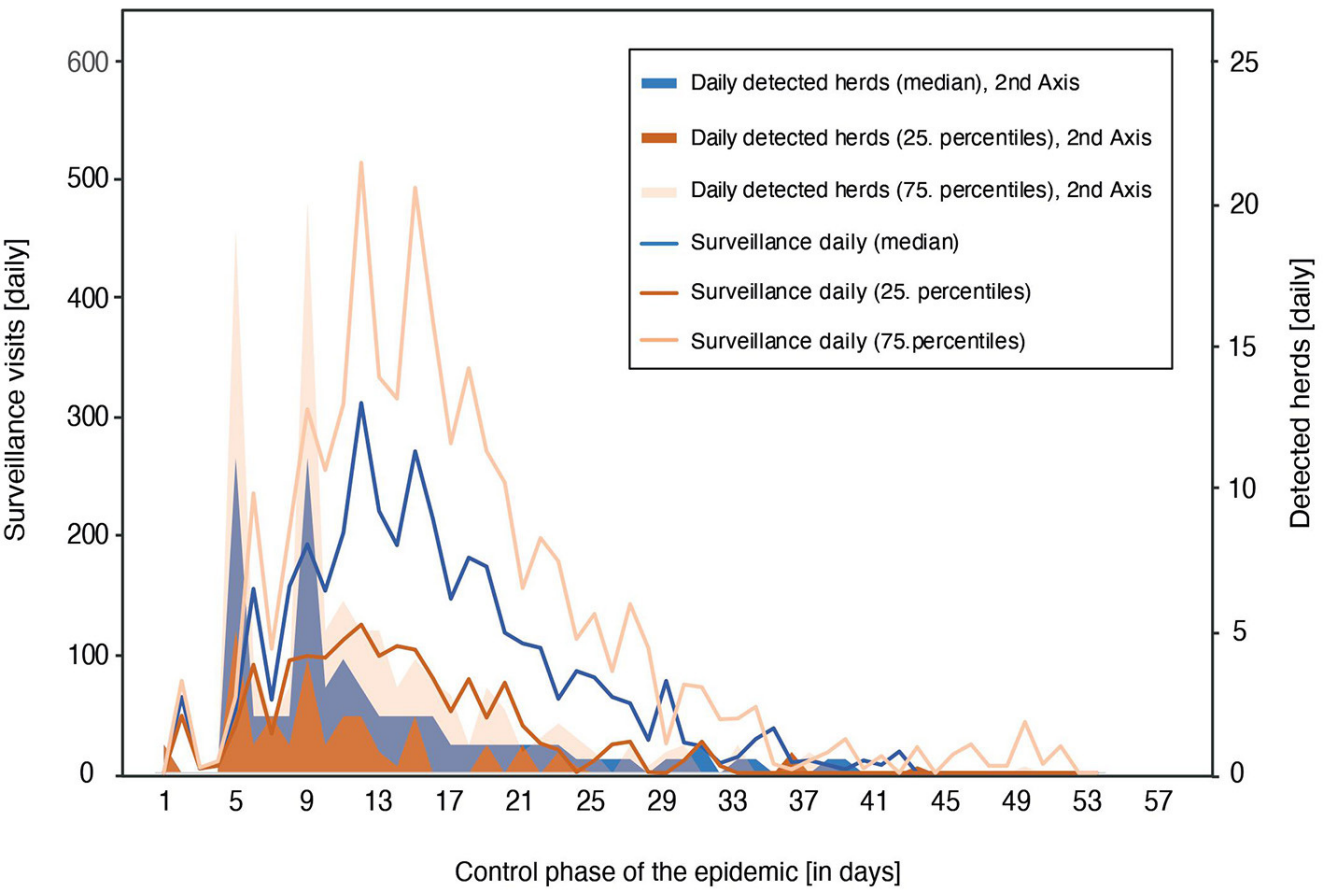
Number of conducted surveillance visits and number of new detected infected herds (2^nd^ axis) in the daily course of the control phase under reference control strategy of an FMD outbreak in Austria.

**Figure 2:**
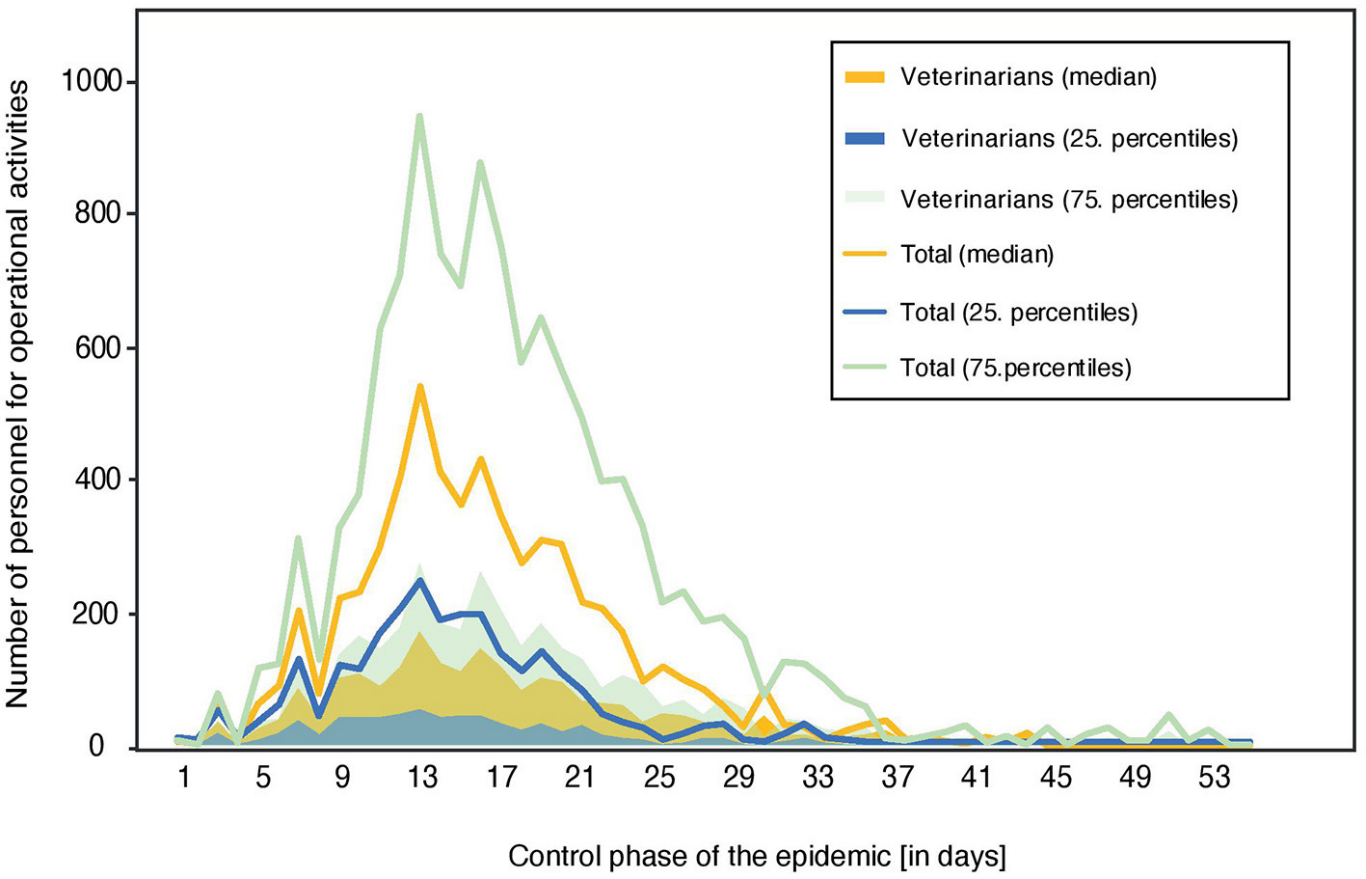
The number of human resources, stratified by veterinarians and total personnel which are required to perform the operational activities in the reference scenario (SO) during the control phase of an FMD epidemic in Austria. N.B. Staffs employed in national and local coordination centers are not included.

Simulations of the reference scenario with unlimited resources resulted in an outbreak size of 61 (27–102) infected herds and 3,837 (1,323–6,400) surveillance visits conducted during the control phase (Figure 1). The highest personnel requirements for surveillance were reached around day 14 with a median of 311 (125–511) staff. Resources for cleaning and disinfection also peaked around this time with a need for 210 (150–382) personnel. Thus, the highest total number of human resources required (for all operational activities) for the optimal response under the reference scenario resulted at the end of the second week with a median of 540 (257–926) personnel, out of which 31% are veterinarians (Figure 2). Additionally, 100 staff (administrative personnel and veterinarians) would be required in the national crisis center and 20 staff in each local coordination center during the control phase of the epidemic. The most effective strategy based on the lowest total costs is the dangerous contact herd depopulation strategy (SODC). In detail, implementation of this control strategy resulted in an outbreak size of 54 (23–97) infected herds, with 3,397 (1,474–6,731) conducted surveillance visits in affected zones. This requires a number of human resources which would peak at the end of the second control week with a median of 300 (117–506). Overall, 470 (210–744) personnel would be needed around this time (14^th^ day), excluding the additional staff in national and local centers.

The pre-emptive depopulation scenario under unlimited resources approach (SORC1) shows increased demand on staff for disposal and cleaning and disinfection with a peak on day 7 (disposal) and 14 (cleaning and disinfection) of the control phase, i.e., a median of 71 (39–111) and 540 (330–1,395) personnel are needed, respectively. Both vaccination control strategies (SOSV1 and SOPV1) require additional staff for the performance of vaccination. In detail, the vaccination strategy would start at the beginning of the second week of the control phase with an immediate peak of required human resources. In total 139 (55–247) and 134 (56–244) herds will be vaccinated and on average, 14 (7-24) and 14 (7-25) staff would be necessary on each day during the first week of vaccination (i.e., second week of control) of the SOSV1 and SOPV1.

**Figure 3:**
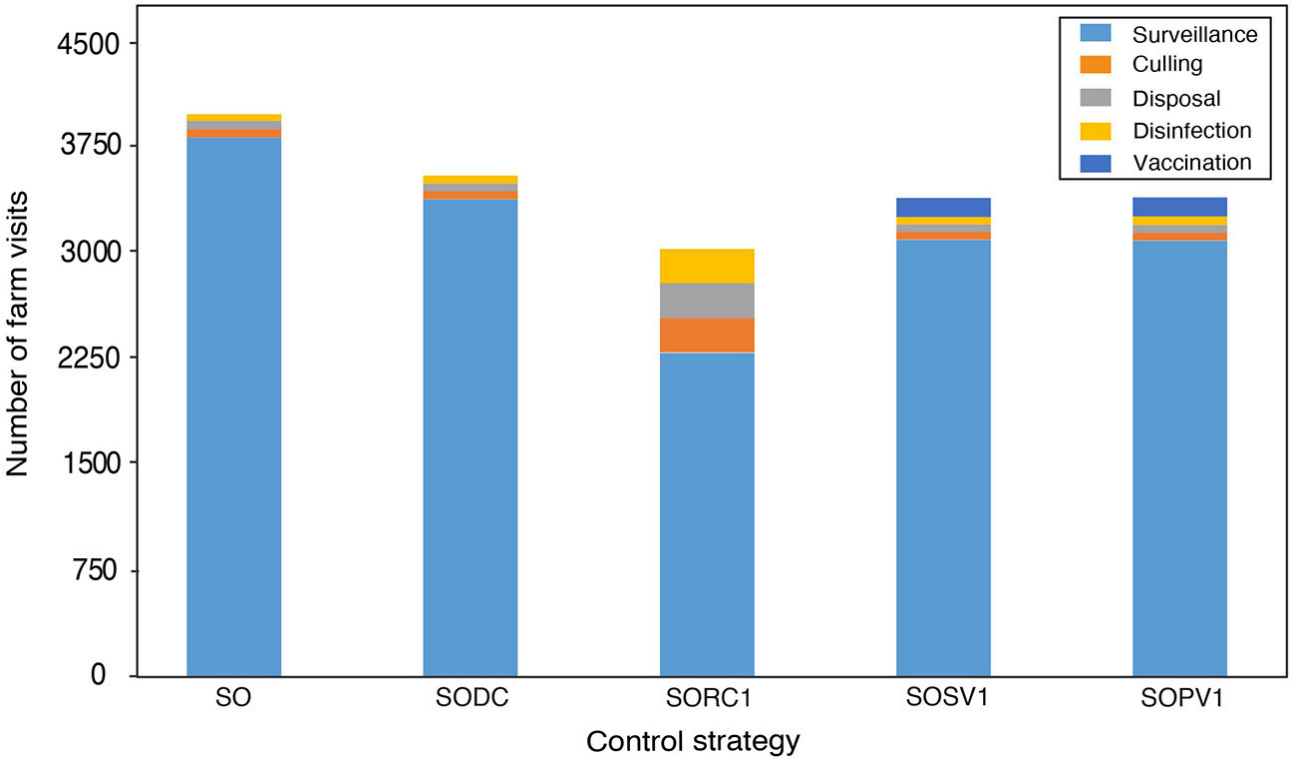
Comparison of overall operational visits to farms split by the type of key operational activities within various control strategies (median values). The considered control strategies are: SO, stamping out of all infected herds (reference scenario); SODC, pre-emptive depopulation of dangerous contact herds; SORC1, pre-emptive depopulation of all susceptible herds within 1 km radius around infected herds; SOSV1, suppressive vaccination of all susceptible herds with 1 km radius around infected herds; SOPV1, protective vaccination of all susceptible herds with 1 km radius around infected herd.

Comparison of the relative effectiveness of different control strategies in terms of the number of overall operational visits shows, that the pre-emptive depopulation strategy (SORC1) would require the fewest operational visits overall: 3,030 (1,340–5,632), a 33% reduction compared to the reference scenario (Figure 3). However, this efficacy comes at the cost of having to depopulate more herds than all other control strategies, four times as many as in the reference scenario.

When simulating with a sufficient amount of resources, the dangerous contact herd depopulation strategy (SODC) proved to be the most optimal control strategy in terms of incurred costs (Table 1). It requires 470 (210–744) personnel on its peak day (14^th^ day), while the highest weekly average of resources needed is reached in the 3^rd^ control week, with 291 (114–518) personnel per day.

## Discussion

The epidemiological and economic analyses in the present study have demonstrated the importance of targeted planning of human resources for a potential FMD outbreak in Austria. To evaluate the personnel requirements in the event of an FMD outbreak, the most livestock-dense region in Austria was chosen as the model region. Epidemics that originate in areas of high livestock density tend to result in larger outbreaks. Previous simulation studies have shown that the median size for outbreaks that begin on dairy farms in this region corresponds to the 65th percentile of the outbreak size for outbreaks that originate on a randomly selected dairy farm. The results of our simulations have shown that sufficient capacity of personnel employed in control measures and operational activities can reduce the total cost of the outbreak by up to 22% and shorten the duration of an outbreak by up to 72%. Even though modeling with unlimited resources reflects an optimistic scenario which does not necessarily correspond to reality, it serves as an orientation in striving to achieve the optimal epidemiological output, lowest possible total cost, and to maximize the effectiveness of the control measures chosen.

The results of our simulations have shown that an outbreak under the reference scenario with unlimited resources would lead to 61 infected herds in the median. If sufficient resources are available, all other scenarios lead to significantly fewer infected farms. Interestingly, in the pre-emptive depopulation control strategy (SORC1), an increase in human resources does not have any impact on the number of infected herds, but it influences the control duration and thus the costs of the outbreak. Among the analyzed scenarios with unlimited resources, the dangerous contact depopulation control strategy (SODC) leads to the lowest total costs and would be the most preferable control strategy. When simulating with constrained resources, as used previously (1), the depopulation control strategy (SORC1) achieved the lowest total costs, but at the expense of thousands of culled animals. Our results have shown that the highest personnel requirements for operational activities would arise at the end of the second control week. This is caused by the fact that at this point, many protection and surveillance zones have to be sampled, but at the same time, a high number of infected herds undergo the disinfection process. These two activities are labor-intensive and, at the time of the peak in the reference scenario, they make up 95% of the total resource requirements. In contrast, Boklund et al. (6) reported in a similar FMD study that the personnel requirements for control of an outbreak in Denmark peaked on the first few days of control. These results differ from our findings presented here also if we take into account that the outbreak simulated in Denmark was of a much smaller dimension (in median 22 infected herds). Our results are in line with the results of Garner (16), who assumed, that the highest number of operational visits such as surveillance, will be necessary in the second control week.

It was not surprising to find out that the success of outbreak control is mainly determined by the capacity of personnel available for surveillance. In our simulated reference scenario, 3,837 (1,323–6,400) surveillance visits were carried out, more than 70% of them in the first 21 days of the control phase. In this context, the question arises as to whether Austria has enough staff to carry out these activities. According to the current report by the Austrian Animal Health Service (17), 771 veterinarians in Austria who offer medical care to livestock farms, have contracts with this organization and would most likely be involved in the performance of operational activities. However, the overall number of farm veterinarians in Austria is, of course, much higher. Approximately 45% of these veterinarians are settled in our model region. This additional capacity would cover even worse-case scenario requirements for surveillance, as there is only necessity for one veterinarian in one surveillance team. However, this assumption does not consider the effect of personnel quarantining which would be necessary between farm visits during the outbreak. We estimated the human resources required on a daily basis and assumed that the same staff would not be deployed throughout the entire control phase. Another critical point identified here is the disposal of the carcasses, as was the case during the UK outbreak in 2001 (10). In Austria, there are two rendering plants that are equipped for disposal in the case of FMD. In standard operation, they process 87,000 tons (t) per year. In the event of an epidemic, the weekly capacity can be increased by a further 3,500 t. This would be sufficient even for the worst-case outbreaks under the reference scenario but might lead to bottlenecks under the pre-emptive depopulation control strategy (SORC1). The last critical point is the cleaning and disinfection of the infected farms where backlogs could arise, especially in scenarios with pre-emptive depopulation. This process is not only staff-intensive (31 staff per team), but also very time-consuming, as the disinfectants have a long exposure time. However, since the teams can be deployed elsewhere during this exposure time and certain preliminary work can also be handed over to farmers, we assume that the actual time spent by the teams on the farm will be shorter. For modeling purposes, we assumed an average value of 1.5 days/operation. According to information from the Austrian armed forces, which support veterinary authorities in the disinfection activities, there are currently enough resources to perform cleaning and disinfection of affected herds during a moderate FMD outbreak in Austria.

For the efficient handling of operational activities, not only the requirements regarding the number of staff but also regarding training and effectiveness of work performance, are of high importance. The necessary duration of each operational activity used in our calculation depends on the type and size of the herd (Supplementary Table 1) and is based on the information from the scientific literature and expert opinions. It is difficult to take into account the differences in the level of education and training of staff in the presented estimates. However, it is very important to provide regular training of veterinarians in relation to outbreaks of animal diseases.

The results estimated in this study are not intended to represent definitive figures on human resource requirements in the event of an FMD outbreak in Austria. Rather, the findings are an assessment of the trend dynamics in staffing requirements needed to respond efficiently to an unexpected outbreak. The past epidemics of FMD in FMD-free countries showed the importance of targeted preparation for such crises (10–13). EuFMDiS is a simulation tool that enables such preparation under realistic conditions. It is one of the few models worldwide that includes economic components and an assessment of resource planning in addition to epidemiological data. This model has been continuously expanded to include new European member countries and features and to cover further animal diseases (e.g., classical swine fever). It is of utmost importance that more participating countries carry out assessments as presented here in order to support the readiness of European countries in the event of cross-border outbreaks. Such estimates also provide useful insights for controlling outbreaks of other unexpected highly contagious animal diseases. Veterinary authorities in Austria should consider the outcome of this study in their contingency planning for FMD. The investments in human resources and their education need to be supported in order to limit the overall negative impact of a potential FMD outbreak in Austria to the best extent possible.

## Data Availability Statement

The raw data supporting the conclusions of this article will be made available by the authors, without undue reservation.

## Author Contributions

TM and BC conceived, designed, and coordinated the study. IK and SS parameterized the model for Austria. TM ran the model and summarized the results. TM, BC, and IK drafted and revised the manuscript critically. All authors contributed to the article and approved the submitted version of the manuscript.

## Funding

This study was partially supported by funding from the European Union's Horizon 2020 Research and Innovation programme under grant agreement number 773830: One Health European Joint Programme (COHESIVE project) and by the Project VET-Austria, a cooperation between the Austrian Federal Ministry of Health, the Austrian Agency for Health and Food Safety and the University of Veterinary Medicine, Vienna, Austria.

## Author Disclaimer

The contents in this manuscript are the work of the authors and do not necessarily reflect the views of the European Commission for the Control of Foot-and-Mouth Disease (EuFMD).

## Conflict of Interest

The authors declare that the research was conducted in the absence of any commercial or financial relationships that could be construed as a potential conflict of interest.

## Publisher's Note

All claims expressed in this article are solely those of the authors and do not necessarily represent those of their affiliated organizations, or those of the publisher, the editors and the reviewers. Any product that may be evaluated in this article, or claim that may be made by its manufacturer, is not guaranteed or endorsed by the publisher.
