# Supplementary material for: What Are the Human Resources Required to Control a Foot-and-Mouth Disease Outbreak in Austria?
Source: Front Vet Sci. 2021 Oct 28;8:727209. doi: 10.3389/fvets.2021.727209 (PMC8580879; doi:10.3389/fvets.2021.727209)
Supplement: Supplementary file 1 [file Data_Sheet_1.docx]

Supplementary Material

# Supplementary Figures and Tables

## Supplementary Figures


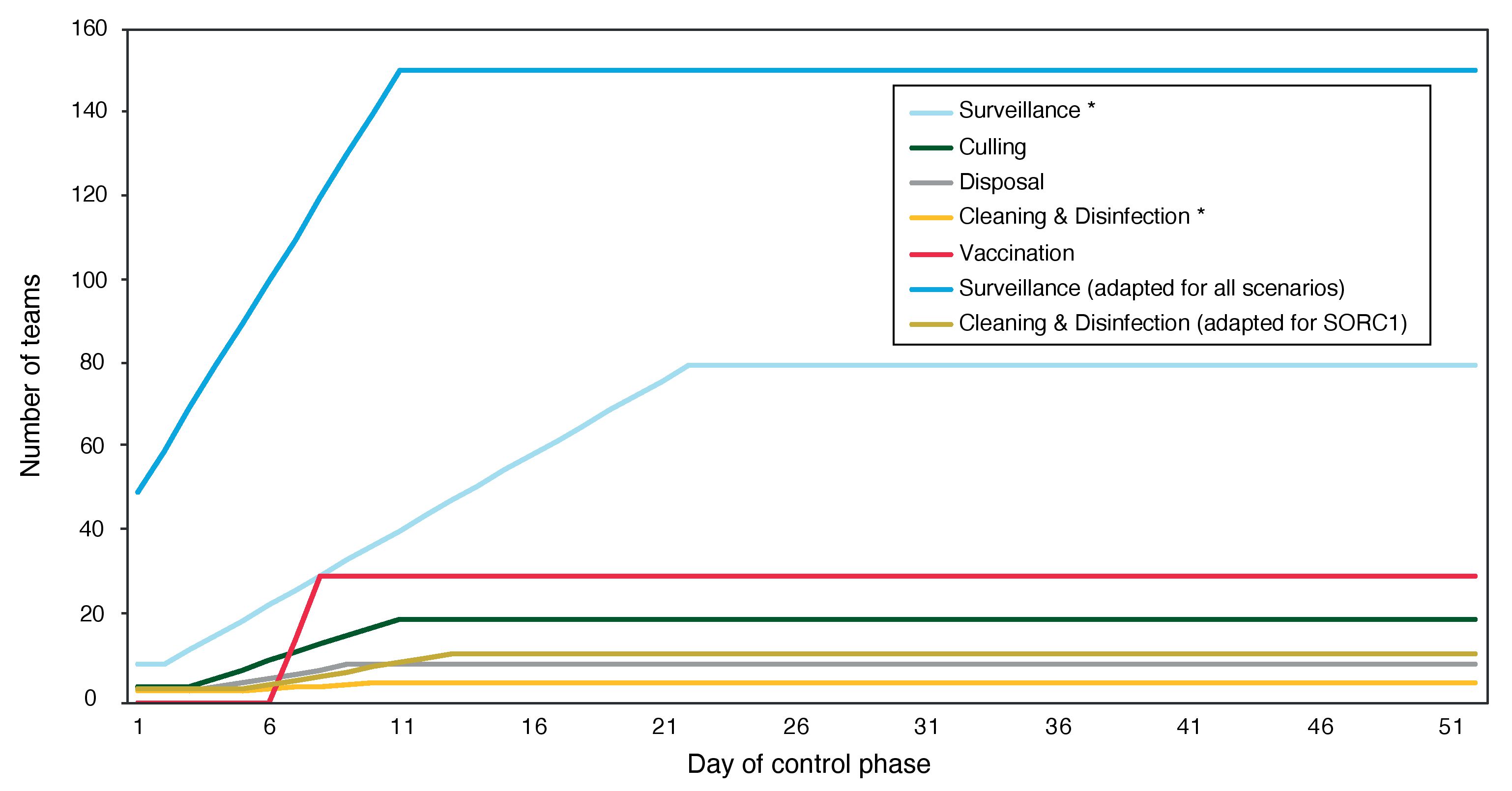


## Supplementary Figure S1. Dynamic allocation of resources in EuFMDiS. Number of teams available for operational activities during an outbreak of FMD in Austria (as used by Marschik et al. (1)).

^*^ Resource capacity for surveillance and cleaning and disinfection was adapted in order to minimize the constraints in the effectiveness of control measures.

## Supplementary Tables

**Supplementary Table S1** Input data regarding duration of operational activities and respective estimation of team size as used in the simulations of FMD outbreak in Austria in EuFMDis

|  | **Surveillance** | **Culling** | **Disposal** | **Cleaning and disinfection** | **Vaccination** |
| --- | --- | --- | --- | --- | --- |
|  |  |  |  |  |  |
| **Herd type** | Duration of control activity in days | | | | |
| Large commercial dairy cattle | 0.5 | 1.0 | 0.5 | 1.5 | 0.4 |
| Large commercial beef cattle | 0.5 | 1.0 | 0.5 | 1.5 | 0.4 |
| Small commercial beef cattle | 0.4 | 0.5 | 0.3 | 1.0 | 0.3 |
| Commercial small ruminants | 0.4 | 0.3 | 0.3 | 1.0 | 0.4 |
| Large-scale commercial fattening pigs | 0.3 | 1.0 | 0.5 | 1.5 | 0.2 |
| Large-scale commercial breeding pigs | 0.3 | 1.0 | 0.5 | 1.5 | 0.2 |
| Small-scale commercial pig | 0.3 | 0.5 | 0.3 | 1.0 | 0.2 |
| Backyard | 0.3 | 0.3 | 0.2 | 0.5 | 0.2 |
|  |  |  |  |  |  |
| Team staff | 1 veterinarian 1 other | 2 veterinarians 4 others | 3 others | 1 veterinarian 30 others | 1 veterinarian 2 others |

*Additionally to operational activities, 100 staff (management, administration, tracing, logistics, press and training) would be necessary in national coordination center and ~ 20 staff/10 detected herds (local coordination, logistics, administration etc.) in local disease control center.*

**Supplementary Table S2.** Input parameters used for calculating the direct costs (€).

| **Herd Type** | **Surveillance** | **Culling** | **Disposal** | | **Compensation** | **Cleaning and disinfection** | **Vaccination** |
| --- | --- | --- | --- | --- | --- | --- | --- |
|  | **(per herd)** | **(per animal)** | **(per animal)** | | **(per animal)** | **(per herd)** | **(per animal)** |
| Large commercial dairy cattle | 6,600 | 50 | 120 | | 1,050 | 3,500 | 2.5 |
| Large commercial beef cattle | 6,700 | 50 | 130 | | 1,050 | 3,500 | 2.5 |
| Small commercial beef cattle | 3,200 | 50 | 120 | | 1,050 | 1,200 | 2.5 |
| Commercial small ruminants | 4,000 | 50 | 12 | | 100 | 1,200 | 2.5 |
| Large-scale commercial fattening pigs | 10,900 | 50 | 20 | | 130 | 1,500 | 2.5 |
| Large-scale commercial breeding pigs | 11,300 | 50 | 20 | | 130 | 1,500 | 2.5 |
| Small-scale commercial pig | 5,100 | 50 | 20 | | 130 | 1,000 | 2.5 |
| Backyard | 930 | 50 | 50 | | 425 | 1500 | 2.5 |
|  | | | |  | |  | |

*N.B. Costs for surveillance, culling, cleaning and disinfection, and vaccination were assessed by the Austrian veterinary authorities. Cost for disposal was obtained from Austrian rendering plants and costs for compensation were derived from the Austrian compensation regulation.*

**Supplementary Table S3.** Input parameters used for calculating the indirect costs (€).

| **Export losses^a^** | | | |  | **Value** | **Source** |
| --- | --- | --- | --- | --- | --- | --- |
|  | Average daily loss of trade to EU countries: | | | |  |  |
|  |  | | Live animals (€) | | 148,978 | (1) |
|  |  | | Livestock products (€) | | 6,260,022 | (1) |
|  | Average daily loss of trade to non-EU countries | | | |  |  |
|  |  | | Live animals (€) | | 87,197 | (1) |
|  |  | | Livestock products (€) | | 963,345 | (1) |
| **Production losses** | | | |  |  |  |
|  | Production losses in zones | | | |  |  |
|  |  | Percentage of lactating cows in a dairy herd | | | 80 | (2) |
|  |  | Average daily milk yield | | | 24.7 | (1) |
|  |  | Price per 1 kg milk (€) | | | 0.4 | (1) |
|  | Production losses in culled herds | | | |  |  |
|  |  | Average daily contribution margin per dairy cow (€) | | | 4.3 | (3) |
|  |  | Average daily contribution margin per beef cattle (€) | | | 0.5 | (3) |
|  |  | Average daily contribution margin per breeding pig (€) | | | 0.1 | (3) |
|  |  | Average daily contribution margin per fattening pig (€) | | | 0.2 | (3) |
|  |  | Average daily contribution margin per small ruminant (€) | | | 1.1 | (3) |

*^a^ Export losses presented here refer only to FMD-susceptible livestock.*

**References (Supplementary Material)**

1. Anonym (Agrarmarkt Austria). Market Report. (2020). Available at: https://www.ama.at/Marktinformationen/Vieh-und-Fleisch/Aussenhandel [Accessed April 25, 2020].

2. Hiesel JA, Kopacka I, Fuchs R, Schobesberger H, Wagner P, Loitsch A, Koefer J. Epidemiological evaluation of different FMD control strategies in two selected regions in Austria. *Berl Munch Tierarztl Wochenschr.* (2016) **129**:484–494. doi:10.2376/0005-9366-15098

3. Anonym (The Federal Institute of Agricultural Economics). Data and Facts - Animal Production. (2020) Available at: https://www.agraroekonomik.at/index.php?id=tierproduktion&L=1&K=0 [Accessed April 23, 2020].
